# Inoculation effects on root-colonizing arbuscular mycorrhizal fungal communities spread beyond directly inoculated plants

**DOI:** 10.1371/journal.pone.0181525

**Published:** 2017-07-24

**Authors:** Martina Janoušková, Karol Krak, Miroslav Vosátka, David Püschel, Helena Štorchová

**Affiliations:** 1 Institute of Botany, The Czech Academy of Sciences, Průhonice, Czech Republic; 2 Institute of Experimental Botany, The Czech Academy of Sciences, Praha, Czech Republic; Friedrich Schiller University, GERMANY

## Abstract

Inoculation with arbuscular mycorrhizal fungi (AMF) may improve plant performance at disturbed sites, but inoculation may also suppress root colonization by native AMF and decrease the diversity of the root-colonizing AMF community. This has been shown for the roots of directly inoculated plants, but little is known about the stability of inoculation effects, and to which degree the inoculant and the inoculation-induced changes in AMF community composition spread into newly emerging seedlings that were not in direct contact with the introduced propagules. We addressed this topic in a greenhouse experiment based on the soil and native AMF community of a post-mining site. Plants were cultivated in compartmented pots with substrate containing the native AMF community, where AMF extraradical mycelium radiating from directly inoculated plants was allowed to inoculate neighboring plants. The abundances of the inoculated isolate and of native AMF taxa were monitored in the roots of the directly inoculated plants and the neighboring plants by quantitative real-time PCR. As expected, inoculation suppressed root colonization of the directly inoculated plants by other AMF taxa of the native AMF community and also by native genotypes of the same species as used for inoculation. In the neighboring plants, high abundance of the inoculant and the suppression of native AMF were maintained. Thus, we demonstrate that inoculation effects on native AMF propagate into plants that were not in direct contact with the introduced inoculum, and are therefore likely to persist at the site of inoculation.

## Introduction

By forming symbiotic association with the majority of terrestrial plants, arbuscular mycorrhizal fungi (AMF) significantly contribute to plant productivity in most ecosystems. They supply their host plants with hardly accessible nutrients, especially with phosphorus, and receive, in return, photosynthetically fixed carbon. In natural conditions, roots of one plant usually become colonized by many AMF species, and the plant's benefits from the symbiosis depend not only on abiotic factors such as soil fertility [1], but also on the infectivity, diversity and composition of the root-colonizing AMF community. For example, AMF communities of high species diversity enhance plant growth more than those of low species diversity [2]. Also, locally adapted AMF communities may be more efficient in providing plant benefits than non-adapted AMF [3–5].

Infectivity and diversity of AMF communities is often reduced in disturbed habitats such as agroecosystems or post-mining sites [6–9]. Adding AMF propagules into soils or pre-inoculation of planted seedlings with AMF has been therefore recommended as a technology to support plant establishment and growth at these sites [10–12]. It is assumed that inoculation effects may be either positive, if the native AMF community is not sufficiently abundant or diverse to provide the maximal mycorrhizal benefits, or negligible, if the native AMF community is sufficient. This assumption, however, disregards potential negative side effects of inoculation due to the inoculants' interactions with native AMF communities.

Inoculation mostly introduces AMF genotypes that are not present at the site of inoculation, and sometimes even originate from different ecosystems or geographical regions [13]. Naturalization and spread of introduced organisms is an important topic of current biology as it may negatively affect biodiversity and ecosystem functioning [14]. Such concerns were also expressed in relation to AMF inoculations, but remain largely hypothetic due to our insufficient knowledge on AMF biogeography, population and community ecology [15]. Inoculation may substantially decrease the diversity of the root-colonizing AMF community by suppressing root colonization by native AMF [10, 16–19]. This suggests low resistance of native AMF communities against the biotic disturbance by introduction of new AMF genotypes. However, only little information is available about inoculation effects exceeding the immediate impact of propagule additions. Inoculated AMF were shown to persist in the roots of the inoculated plants two years post inoculation [10, 20–21]. We ignore, however, whether they are able to spread into root systems of newly emerging seedlings that were not in direct contact with the introduced propagules. Furthermore, we know very little about the resilience of AMF communities following inoculation-induced changes. Effects of inoculation on AMF abundances may disappear within the life cycle of a plant [22], which indicates that inoculation-induced changes in AMF diversity or community composition may be only transient, consistently with the suggestion that plants may actively promote the diversity of their symbionts [23]. On the other hand, AMF community richness may remain severely reduced throughout the whole vegetation season, and only partly restore even two years post inoculation [10]. Further time-course studies and studies employing quantitative molecular tools are therefore needed to describe the abundance dynamics of inoculated isolates and dynamics of AMF communities post inoculation.

Assessing the establishment of inoculants and effects on native AMF is also complicated by considerable genetic and functional diversity encountered within AMF species [24–27]. Inoculation is usually performed with wide-spread species of Glomeraceae family such as *Rhizophagus irregularis* or *Funneliformis mosseae* [13], which are regularly present at the target sites. Inoculation then introduces new intraspecific genotypes that can possess different traits than the conspecific natives. Species-level markers document abundance of the inoculated species [28], but do not provide information about the proportion of the inoculated and native genotypes. This information, however, is essential for a complete picture of inoculation effects.

In order to gain deeper insight into possible longer-term effects of inoculation into soils containing native AMF, we designed a greenhouse experiment addressing two questions: i) Is an inoculant able to spread into root systems of neighboring plants that were not in direct contact with the originally added inoculum? ii) How does inoculation affect the abundances of native AMF in the roots of the directly inoculated plants and their neighbors? The experiment was based on soil from a post-mining site and its native AMF community, i.e. on a typical target system for inoculations. It encompassed two host plants belonging to different functional groups in order to assess the role of host-specific factors, and two modes of inoculation—pre-inoculation of seedlings and inoculation in-situ by placing the inoculant's propagules into soil. In addition to evaluating the AMF communities in roots, we measured the biomass of the experimental plants in order to evaluate possible changes in the plant-growth promoting effects of the AMF communities. We expected that immediate inoculation effects would be more pronounced after pre-inoculation due to priority effects [29–30] and hypothesized that the inoculant would spread into adjacent root systems, but its abundance and the impact on native AMF would decrease in comparison with the directly inoculated root systems.

## Materials and methods

### Experimental system

The experiment was based on the soil-fungi system of the coal mine spoil bank Merkur near Chomutov, North Bohemia, Czech Republic. The site was selected as a representative of a system where inoculation with AMF has been tested within the reclamation process [20, 31]. The site does not belong to any protected area nor was the study concerned with protected species. Access to the site and substrate collection were permitted by Severočeské doly a.s. company responsible for the reclamation of the state-owned land. Grey Miocene Clay forming the surface of the spoil bank was collected from an about 10-year-old site after removal of ruderal vegetation, and homogenized with sand in the ratio of 4:1 (v:v). The chemical parameters of the collected clay substrate were described by [31], the mixture with sand had the following main parameters: pH_(H2O)_ 7.5, C_org_ 1.15%, N 0.06%, Olsen-P (0.5 M NaHCO_3_ extractable) 2.93 mg kg^-1^.

Inoculation was performed with an isolate of *Rhizophagus irregularis* (Błaszk, Wubet, Renker & Buscot) C. Walker & Schuessler (2010) termed ‘Chomutov’. This isolate originated from a different part of the spoil bank than the substrate used for the experimental cultivation, and had been kept in culture for about three years prior to the establishment of the experiment. It was selected, because it could be distinguished from the native *R*. *irregularis* population of the cultivation substrate by specific primers in the large subunit of mitochondrial ribosomal DNA (mtLSU, see subchapter 'Design and optimization of qPCR assays' for details). At the same time, it was expected to be adapted to the conditions of the substrate.

The experiment was performed with two host plant species: medic (*Medicago sativa* L. cv. Vlasta) and reed canarygrass (*Phalaris arundinacea* L. cv. Palaton S). Both taxa were tested for agricultural reclamation of spoil banks [31] and represent two plant functional groups (legume and grass).

### Design and optimization of qPCR assays

The native AMF community was characterized according to partial sequences of the large subunit of nuclear ribosomal DNA (nrLSU) as described in detail in S1 Text. This DNA region was selected due to available primers for universal amplification of all glomeromycotan taxa [32] and because it enables the design of primers for the specific amplification of species-level clades [33]. The population of the native *R*. *irregularis* clade was characterized according to partial sequences of mitochondrial ribosomal DNA (mtLSU) as detailed in S1 Text. In contrast to nrLSU, mtLSU haplotypes of *R*. *irregularis* enable to distinguish intraspecific genotypes of this species [34–35], but on the other hand, the lack of sequence information for most of the other glomeromycotan taxa makes this region unsuitable for the characterization of the whole AMF community. Thus, the effects of inoculation were followed using two primer sets: 1) primers based in the nrLSU targeting species-level clades of the AMF community; 2) primers based in the mtLSU discriminating between the inoculant and conspecific native genotypes.

Phylogenetic analyses of the nrLSU of the native AMF community revealed five clades (S1 Fig), three of which corresponded to the species *Rhizophagus irregularis* (Błaszk, Wubet, Renker & Buscot) C. Walker & Schuessler (2010), *Funneliformis mosseae* (T.H. Nicolson & Gerd.) C. Walker & Schuessler 2010 and *Claroideoglomus claroideum* (N. C. Schenck & G. S. Sm.) C. Walker & Schuessler (2010). Primers designed previously to discriminate isolates of these three species [22, 35] were tested using plasmid standards and root samples and quantified reliably these clades of the native community. Additionally, primers were designed to specifically amplify genotypes of the other two clades of the native community, the ‘uncultured Glomeraceae’ clade and *Diversispora celata* clade C. Walker, Gamper & Schuessler (2009) based on the complete sequence alignment using Primer 3 Plus [36]. The preparation of plasmid standards, qPCR and the estimate of amplification efficiencies generally followed previously described steps [35]. Details on annealing temperatures, primer concentrations, amplicon lengths and the estimated amplification efficiencies for each used qPCR assay are summarized in S1 Table.

Alignments of the *R*. *irregularis* mtLSU sequences revealed three haplotypes (S2 Fig) that could not be completely distinguished by specific primers. However, all of them were amplified by the primer combination GI-PH5-mtLSU-219F and GI-PH5-mtLSU-327R designed to discriminate the *R*. *irregularis* isolate PH5 from isolate ‘Chomutov’ in a previous study [35]. The latter isolate, used as inoculant in this study, was therefore amplified with the same primer combination as in the previous study [35] after confirming that no cross-amplification occurred with genotypes of the native AMF community of the spoil bank soil using plasmid standards and DNA extracts from roots. Details on the used qPCR assay are summarized in S2 Table.

### Cultivation experiment

Inoculum of *R*. *irregularis* 'Chomutov' was prepared by mixing, homogenizing and air-drying substrate from four seven-month-old cultures of the isolate in a mixture of zeolite and sand (1:1) and medic (*Medicago sativa*) as host plant. The cultures were checked microscopically for the presence of spores and absence of contamination. The infectivity of the inoculum and the non-sterile experimental substrate (AMF substrate) were determined by the most probable number test before the experiment as previously described [22] with medic as host plant. The inoculum had an inoculation potential of 55 infective propagules (IP) ml^-1^ substrate, while the inoculation potential of the spoil bank substrate was only 5 IP ml^-1^.

Plants of both species were germinated in autoclaved sand and pre-cultivated in seedling trays for three weeks in a 1:1 mixture of autoclaved zeolite and sand. One third of the seedlings of each species was inoculated with *R*. *irregularis* 'Chomutov' during this pre-cultivation stage by amending the pre-cultivation substrate with inoculum to 30% (v:v) while the other two thirds of the seedlings were left without inoculation. Before the planting of the experiment, roots of five randomly selected inoculated seedlings per plant species were stained with trypan blue and confirmed, using a stereomicroscope, to have at least 30% of their roots colonized with the AMF. Further five randomly selected inoculated and non-inoculated seedlings per species were washed, dried at 65°C and weighted to document possible effects of the inoculation on seedling growth.

The experiment was established in plastic pots (12 × 12 × 9 cm), each divided into two equal compartments (6 × 12 × 9 cm) by a nylon mesh with mesh diameter 42 μm to exclude root competition but enable the spread of extraradical mycelium (ERM) between the compartments. Both compartments were filled with the mixture of spoil bank substrate and sand specified in 'Experimental system'. This substrate either contained its native AMF community ('AMF substrate') or was sterilized by γ irradiation ('control substrate') and both compartments contained the same substrate variant. These two substrate treatments were factorially combined with the two host plant species (*M*. *sativa*, *P*. *arundinacea*), and within each combination of plant and substrate, three inoculation treatments were established as follows.

One seedling, further termed the donor (D) plant, was planted at the start of the experiment into one of the two compartments, while the second compartment remained empty at the start of the experiment. One inoculation treatment (pre-inoculation) was established with the seedlings inoculated during the pre-cultivation stage as described above. In the second inoculation treatment (in-situ), seedlings pre-grown in sterile substrate received inoculation with *R*. *irregularis* 'Chomutov' directly at planting with inoculum mixed into the cultivation substrate of the planted compartment to 4% (v:v). The third inoculation treatment (non-inoculated) was established with seedlings pre-grown in sterile substrate and without additional inoculation. In order to decrease differences in microbial conditions among the experimental treatments, the planted compartments were irrigated with 10 ml of bacterial filtrate obtained by passing a suspension from the AMF substrate and the inoculum through filter paper (Whatman No.1).

**Fig 1:**
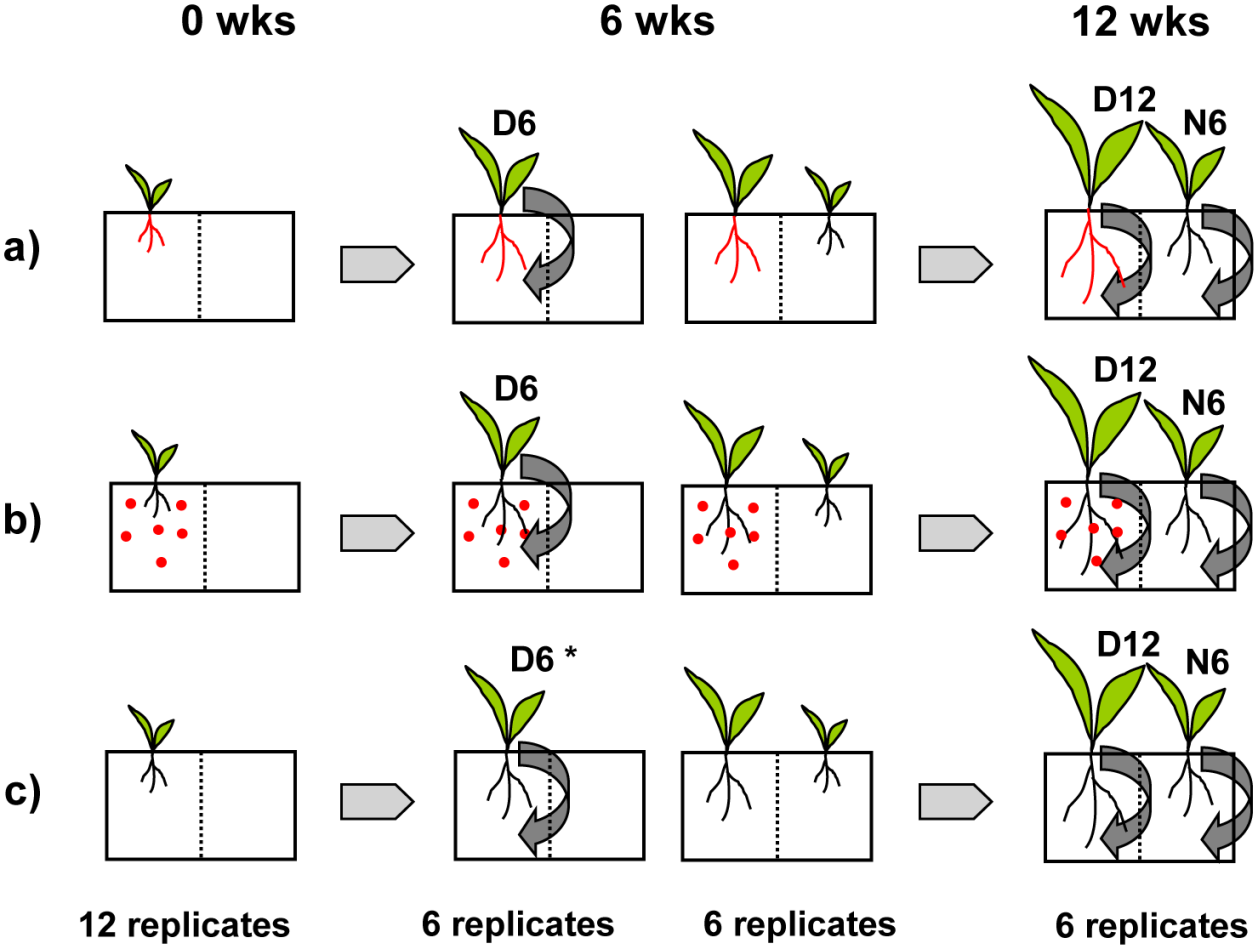
Schematic presentation of the core experimental arrangement. At the start of the experiment (0 wks), the seedlings were either pre-inoculated (a), inoculated with propagules mixed into the cultivation substrate (b) or left without inoculation (c). After six weeks of cultivation (6 wks), six replicates of each treatment were harvested to obtain six-week-old donor plants (D6), the remaining six replicates were planted with a second seedling without any additional inoculum addition. After further six weeks of cultivation (12 wks), the harvest of the remaining replicates rendered 12-week old donor plants (D12) and six-week old neighboring plants (N6). This core arrangement was established four times: with two plant species (*M*. *sativa* and *P*. *arundinacea*), each either in substrate containing its native AMF community or in sterilized control substrate. The asterisk denotes replicates that were not established in the sterilized control substrate.

Each combination of substrate, plant and inoculation treatment was established in 12 replicates (except for the non-inoculated treatments in control substrate, which were established in six replicates only). After six weeks of cultivation, the second compartments of six pots per treatment were planted with a second plant of the same species, termed the neighboring (N) plant, without any additional inoculation. The other six pots per treatment were harvested to evaluate the growth and AMF community of the six-week-old D plants. The pots with both compartments planted were harvested after further six weeks of cultivation, i.e. when the D plants were 12 weeks old and the N plants six weeks old (Fig 1). Thus, each combination of the three factors plant species, substrate and inoculation treatment comprised three 'stages': the six-week-old D plants, twelve-week-old D plants and six week old N plants. The only exception was the non-inoculated treatment in control substrate, which was established in six replicates only and did not include six-week-old D plants (see Fig 1).

The experiment was cultivated in a glasshouse under supplemental light (12 hours, metalhalide lamps, 400 W) and fertilized once in two weeks with 50 ml of P2N3 nutrient solution per plant [37]. In some replicate pots, the N plants did not properly establish in the experimental system and died back. These replicate pots were completely excluded from the harvest and further evaluations with the consequence that replicate numbers were reduced in some treatments as specified in S12 Table.

### Harvest and data collection

Each root system was washed, weighed and cut into 1 cm segments. A subsample of 100 mg fresh weight was flash frozen in liquid N and stored at -80°C. Another part was used for microscopic determination of root colonization after staining with 0.05% trypan blue in lactoglycerol [38]. The remaining roots and shoots were dried at 65°C for 24 h and used for the determination of shoot and root dry weights. Percentage of root colonization by AMF was determined as frequency of AMF structures in root segments corresponding in length to the diameter of the microscopic field (×100 magnification; Olympus BX60).

Genomic DNA was extracted from the deep-frozen samples using the DNeasy Plant Mini Kit (Qiagen) according to the manufacturer’s instructions. DNA extracts from root samples were quantified spectrophotometrically, and 10 ng of total genomic DNA were used as a template in qPCR as previously described [36]. All harvested root systems per treatment and harvest were analyzed. All target sequences were quantified in the root samples from the AMF substrate. The abundances of the AMF taxa were calculated as copy numbers per ng of template DNA.

### Data analysis and statistics

Variation in root colonization was statistically analyzed for each substrate separately using three-way ANOVA with the factors plant species, inoculation and stage after arcsine transformation of the data. The non-inoculated treatment in control substrate with overall zero root colonization was excluded from the analysis.

Variation in shoot biomass was assessed for each plant species and stage separately; one-way ANOVA followed by Tukey's test was used to test for differences between each combination of substrate and inoculation treatment.

The abundance (= mtLSU copy numbers) of the inoculant was statistically analyzed using four-way-ANOVA with the factors substrate, plant species, inoculation and stage. The non-inoculated treatment was excluded from the analysis. Proportion of the inoculant at the total *R*. *irregularis* population in AMF substrate, which was calculated as the sum of mtLSU copy numbers of the inoculant and the native genotypes, was analyzed by three-way-ANOVA with the factors plant species, inoculation and stage after arcsine transformation of the data. The same approach was used to assess the variability in the abundance (= mtLSU copy numbers) of the native *R*. *irregularis* population, but including also the non-inoculated treatment into the analysis.

The composition of the AMF community in AMF substrate was assessed based on nrLSU copy numbers of the species-level clades without distinguishing between the inoculant and native genotypes within the *R*. *irregularis* clade. As all samples contained the same four AMF taxa, the composition of the AMF community was described by Pielou's evenness index. The index was calculated based on Shannon's diversity index as J' = H'/H'max, where H' is Shannon's diversity index and H'max = ln (4). The variability in J' and in the abundance of each taxon was evaluated by three-way ANOVA with the factors plant species, inoculation and plant stage. The abundances of the other AMF taxa than *R*. *irregularis* were also summed to 'abundance of the non-inoculated taxa', which was analyzed in the same way.

The ANOVAs were calculated in STATISTICA (version 12, StatSoft, Inc., USA). When necessary, the abundance data were square-root or logarithmically transformed to fulfill the assumption of ANOVA on homogeneity of variance (tested by Levene's test). Multiple comparisons were performed using Tukey's test.

Additionally, AMF community composition was analyzed by multivariate analyses in CANOCO [39] (version 5.0). Redundancy Analysis (RDA) was performed with non-transformed data 'centered and standardized by species'. The significance of the effects was tested using a Monte Carlo test with 499 permutations.

### Accession numbers

Representative sequences of partial nrLSU of the native AMF taxa were submitted to GenBank under the accession numbers KC537331–360. Partial mtLSU sequences of native *R*. *irregularis* haplotypes were submitted under the accession numbers KC537361–363.

## Results

### Root colonization by AMF

In AMF substrate, root colonization ranged between 52% and 83% (average value per treatment, S3 Table) and was significantly higher in *P*. *arundinacea* than in *M*. *sativa*. Root colonization was also affected by the factor stage and the interaction of stage and inoculation (S4 Table) in AMF substrate, but there were no significant differences among the inoculation treatments within each stage.

No root colonization was found in the non-inoculated plants in control substrate. The inoculated plants in control substrate had overall higher root colonization than plants growing in AMF substrate (F_(1,168)_ = 19.619, P < 0.001). Similarly as in AMF substrate, root colonization was significantly higher in *P*. *arundinacea* than in *M*. *sativa*, and affected by the factor stage and the interaction of stage and plant species; inoculation had no effect, i.e. the pre-inoculated and in-situ inoculated plants did not significantly differ in root colonization (S4 Table).

### Establishment of the inoculant and response of native *R*. *irregularis* genotypes

The abundance of the inoculant *R*. *irregularis* Chomutov, determined as mtLSU copy numbers, was significantly higher in control substrate than in AMF substrate; the significant interaction of the factors substrate and plant species reflects that the difference was more pronounced in *P*. *arundinacea* than in *M*. *sativa* (S5 and S7 Tables). Inoculation or stage had no effect on the abundance of the inoculant (S5 Table).

**Fig 2:**
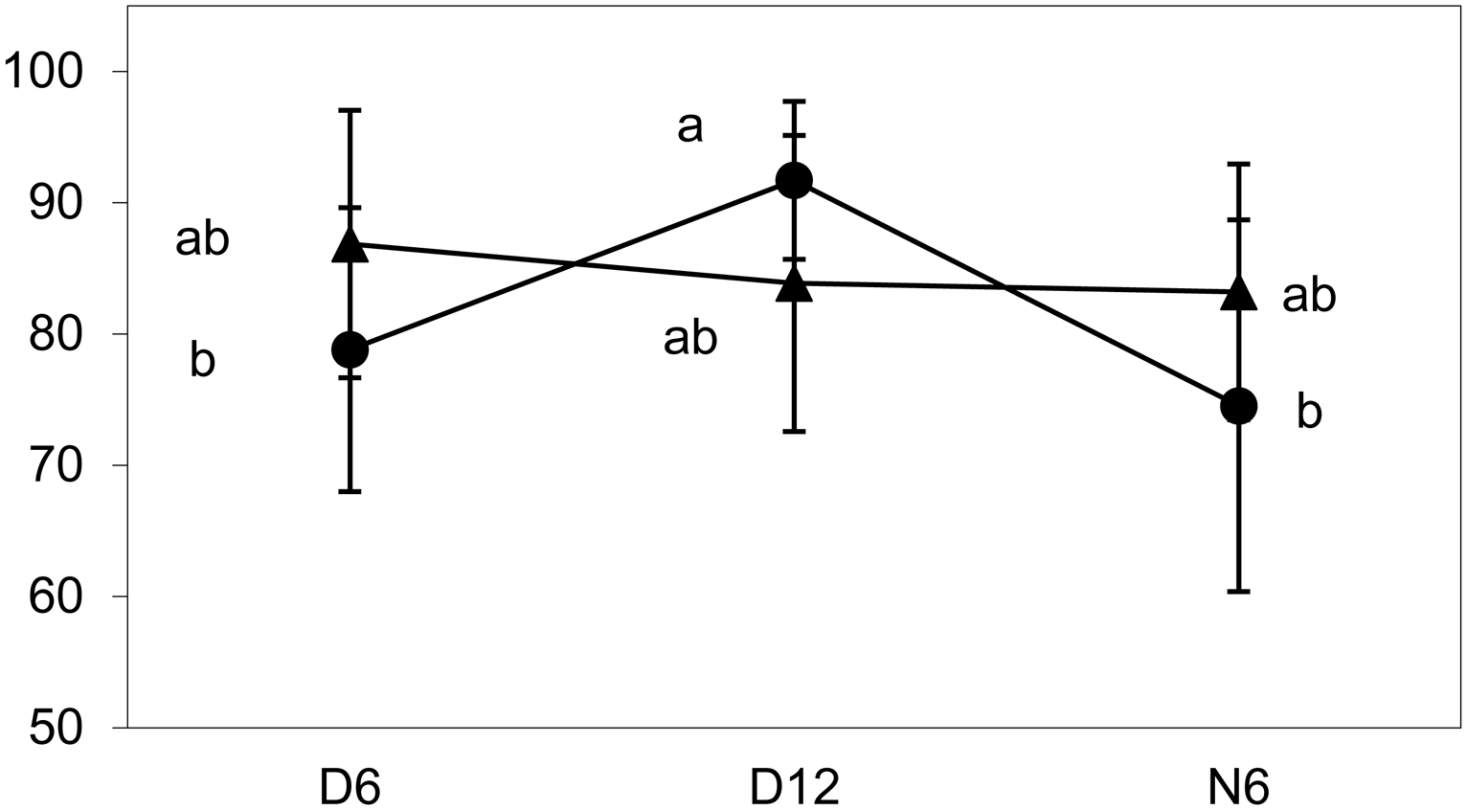
Proportion of inoculated *R*. *irregularis* Chomutov at the *R*. *irregularis* population. Triangles and circles refer to the pre-inoculated and in-situ inoculated treatment, respectively. D6 and D12 are directly inoculated donor plants harvested after six or 12 weeks of cultivation, respectively; N6 are six-week-old neighboring plants. Data are pooled for the two plant species *M*. *sativa* and *P*. *arundinacea*, each symbol represents the mean of 9–12 replicates (± SD), for exact replicate numbers see S12 Table. Means significantly different at P < 0.05 according to Tukey's test are marked by different letters.

In the inoculated treatments in AMF substrate, the proportion of the inoculant at the total *R*. *irregularis* population ranged between 76 and 92% (average per treatment). The proportion was significantly higher in *M*. *sativa* than in *P*. *arundinacea* and varied among the stages depending on the form of inoculation (Fig 2, S6 Table). After in-situ inoculation, the proportion was significantly higher in 12-week-old than in six-week-old D plants, but it did not differ between 6-week-old D and N plants.

**Fig 3:**
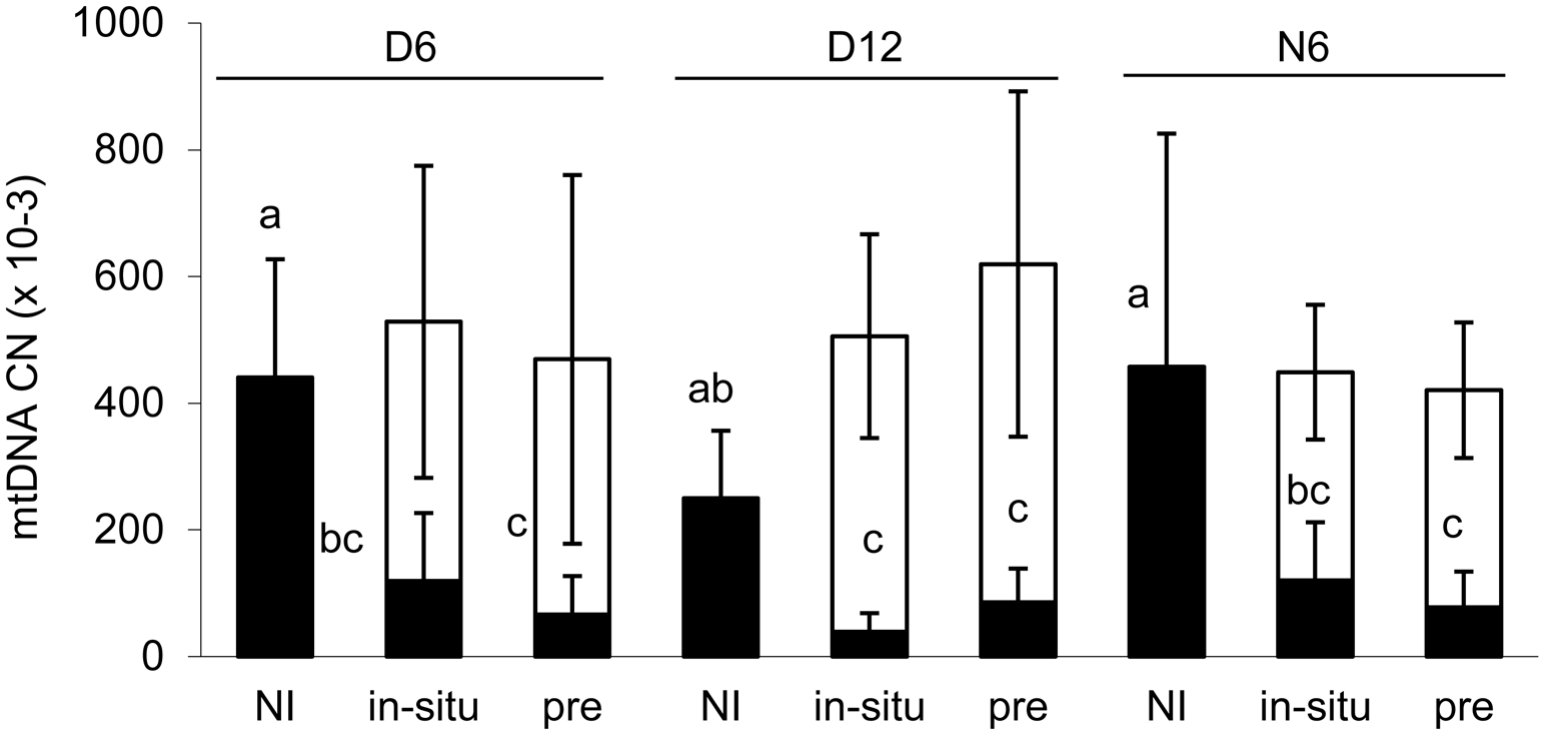
Abundance of native *R*. *irregularis* genotypes and the inoculant *R*. *irregularis* Chomutov. Black boxes and empty boxes show copy numbers of mitochondrial ribosomal DNA of the native genotypes and the inoculant, respectively, in the roots of plants cultivated without inoculation (NI), inoculated in-situ (in-situ) or preinoculated (pre) with *R*. *irregularis* 'Chomutov'. D6 and D12 are directly inoculated donor plants harvested after six or 12 weeks of cultivation, respectively; N6 are six-week-old neighboring plants. Data are pooled for the two plant species *M*. *sativa* and *P*. *arundinacea*, each box is the mean of 9–12 replicates (± SD), for exact replicate numbers see S12 Table. Letters refer to the black boxes, means significantly different at P < 0.05 according to Tukey's test are marked by different letters.

The abundance of the native *R*. *irregularis* genotypes was significantly affected by inoculation, stage and the interaction of both factors (S6 Table). In-situ inoculation and pre-inoculation significantly decreased their abundance throughout the experiment as compared to the non-inoculated treatment; the interaction consisted in the varying size of the effect (Fig 3, S7 Table).

### Effect of inoculation on the species-composition of the AMF community

**Fig 4:**
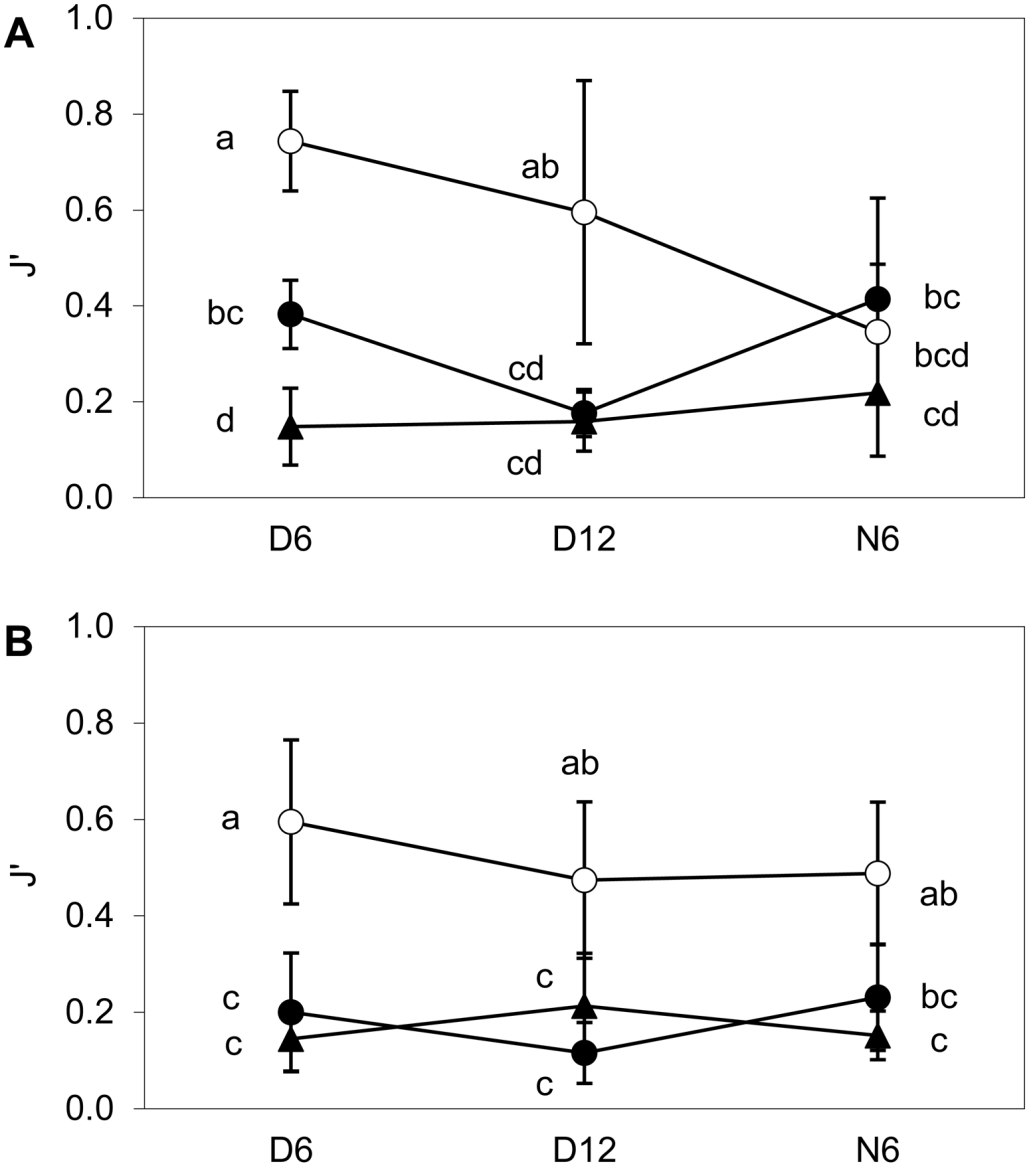
Pielou's evenness index J' of the arbuscular mycorrhizal fungal communities. Roots of (A) *M*. *sativa* and (B) *P*. *arundinacea*, each either without inoculation (empty circles), inoculated in-situ (full circles) or pre-inoculated (full triangles) with *R*. *irregularis*. D6 and D12 are directly inoculated donor plants harvested after six or 12 weeks, respectively; N6 are six-week old neighboring plants. Each symbol represents the mean of 4–6 replicates (± SD), for exact replicate numbers see S12 Table. Means significantly different at P < 0.05 according to Tukey's test are marked by different letters.

The *Diversispora celata* clade could not be detected by the specific qPCR system in any of the experimental samples. The remaining four species-level clades were found in all root samples from AMF substrate so that the species richness of the root-colonizing AMF community was unaffected by inoculation. However, inoculation significantly affected the evenness index J' of the community, the effect depended on stage and plant species (S8 Table). Specifically, pre-inoculation and in-situ inoculation decreased J', as compared to the non-inoculated treatment, in the D plants of both plant species, while J' was unaffected by inoculation in the N plants of *M*. *sativa* (Fig 4A) and significantly decreased by pre-inoculation in the N plants of *P*. *arundinacea* (Fig 4B).

**Fig 5:**
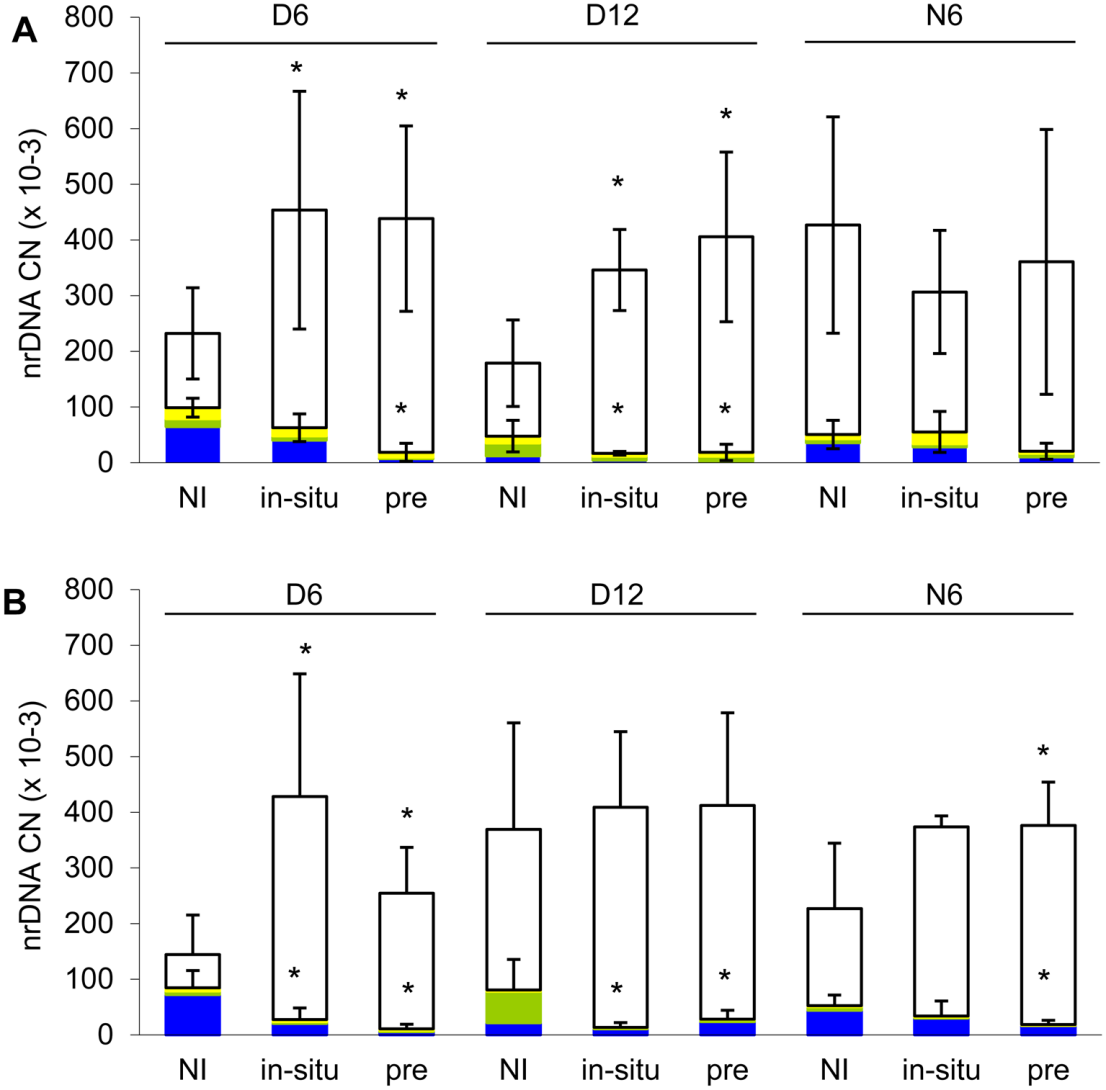
Abundance of each taxon within the arbuscular mycorrhizal fungal communities. (A) Roots of *M*. *sativa*, (B) roots or *P*. *arundinacea*, each either without inoculation (NI), inoculated in-situ (in-situ) or pre-inoculated (pre) with *R*. *irregularis*. Boxes show copy numbers of nuclear ribosomal DNA, empty boxes refer to *R*. *irregularis*, the other, non-inoculated taxa are distinguished by colors (*F*. *mosseae*—blue, *C*. *claroideum*—green, uncultured Glomeraceae—yellow). D6 and D12 are directly inoculated donor plants harvested after six or 12 weeks, respectively; N6 are six-week old neighboring plants. Boxes are means of 4–6 replicates, for exact replicate numbers see S12 Table. Vertical lines show SD for the abundance of *R*. *irregularis* or the sum of abundances of the non-inoculated AMF taxa. Asterisks refer to the same values as the SD and indicate, within D6, D12 or N6, significant difference from the non-inoculated treatment at P < 0.05 according to Dunnett's test.

The effect of inoculation on community evenness J' was mainly due to more pronounced dominance of *R*. *irregularis* in the inoculated treatments. Inoculation generally increased the abundance of *R*. *irregularis* and decreased the sum of abundances of the other AMF taxa. The effect of inoculation, however, depended on plant stage and, in the case of the other AMF taxa, also on plant species (S8 Table). To summarize comparisons among inoculation treatments performed for each stage separately (Fig 5), inoculation mostly affected the abundance of *R*. *irregularis* and the non-inoculated taxa in D plants and mostly had no effect in N plants. The abundances of each of the non-inoculated AMF taxa, *F*. *mosseae*, *C*. *claroideum* and the uncultured Glomeraceae clade, and their responses to the experimental factors are given in S9 and S10 Tables.

**Fig 6:**
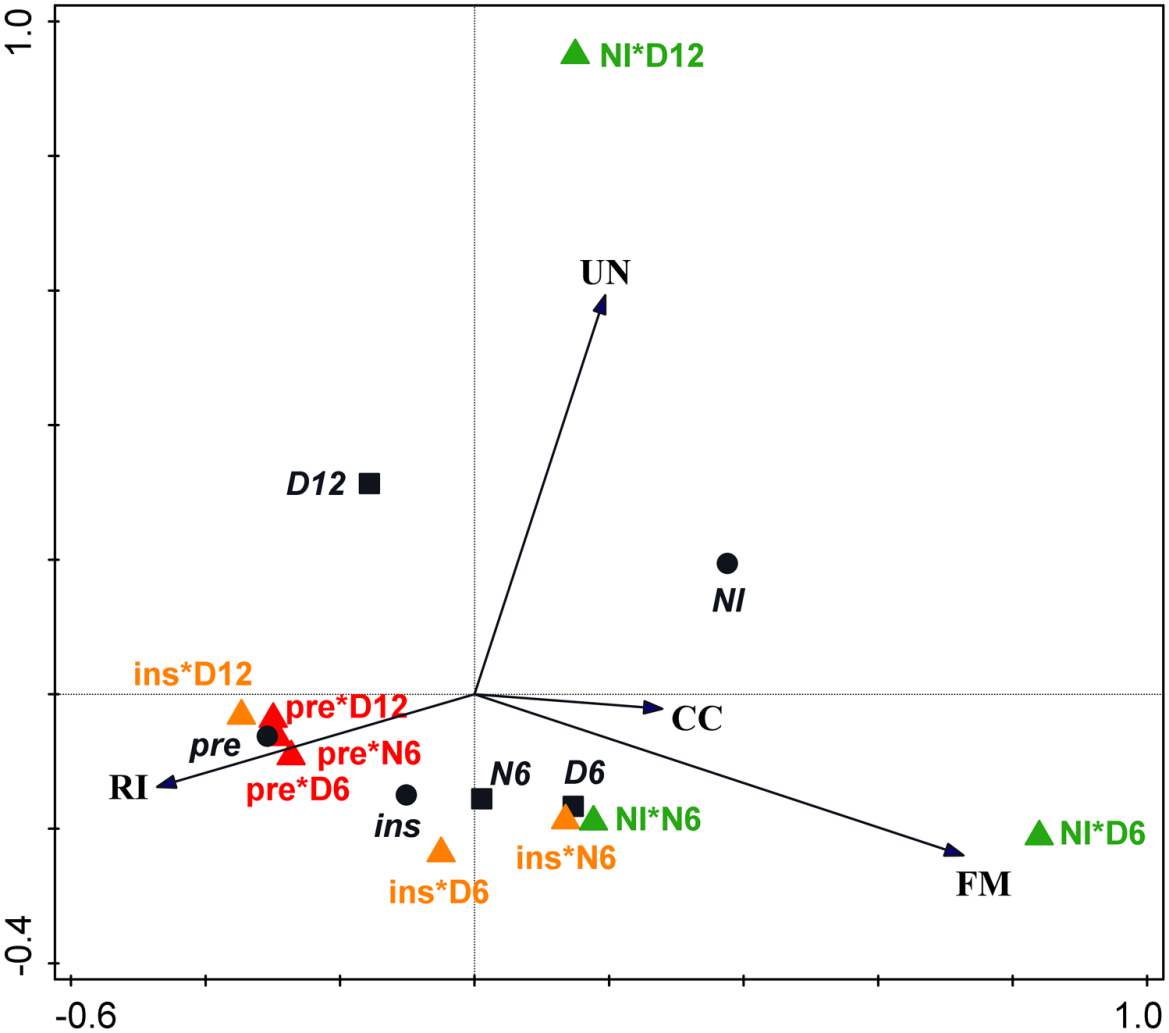
Redundancy analysis of the arbuscular mycorrhizal fungal communities. The model (pseudo F = 6.6, P = 0.002) included the experimental factors inoculation and stage and their interaction; plant species was added as covariate. It accounted for 36.8% of the variability in the data (22.7% is explained by the first axis and 11.2% by the second axis). Black arrows represent taxa of the arbuscular mycorrhizal fungal communities (RI—*R*. *irregularis*, FM—*F*. *mosseae*, CC—*Claroideoglomus claroideum*, UN—uncultured Glomeraceae), black circles are inoculation treatments (NI—non-inoculated, ins—inoculated in-situ, pre—pre-inoculated), black squares are stages (D6–6-week-old donor plants, D12–12-week-old donor plants, N6—neighboring plants), triangles in colors are combinations of inoculation and stage.

The variation in AMF community composition data was explained by the three experimental factors by 24.5% (pseudo F = 7.4, P = 0.002). Inoculation accounted for the highest proportion of the explained variation (15.6%), followed by stage (6%) and plant species (4%). RDA with the factor plant species as covariate confirmed, in separate models, significant effects of inoculation (pseudo F = 10.9, P = 0.002), stage (pseudo F = 4.8, P = 0.004) and interaction of the two factors (pseudo F = 4.1, P = 0.002). Model including both factors and their interaction (pseudo F = 6.6, P = 0.002), with plant species as covariate, revealed close association of *R*. *irregularis* with the pre-inoculated treatment and similarity of the AMF communities after pre-inoculation in all three stages (Fig 6). In contrast to the pre-inoculated treatment, the composition of AMF communities without inoculation was variable in the three plant stages, with relatively high abundance of *F*. *mosseae* in six-week old D plants and the uncultured Glomeraceae clade in 12-week old D plants.

### Plant growth

At the time of their planting into the experiment, the pre-inoculated seedlings of *P*. *arundinacea* were significantly smaller than the non-inoculated seedlings (*t* = -2.97, *P* = 0.02, S11 Table). The same trend, marginally non-significant, was observed in *M*. *sativa* (*t* = -2.20, *P* = 0.06, S11 Table). In the experiment, *M*. *sativa* had the smallest biomass when non-mycorrhizal, i.e. in the non-inoculated treatment in control substrate (Table 1). Six-week old D plants of *M*. *sativa* were significantly smaller after in-situ inoculation than after pre-inoculation or with native AMF only. Twelve-week old D plants of *M*. *sativa* had the highest biomass when grown pre-inoculated in control substrate, and the biomass of N plants did not differ among the experimental treatments except for the smallest biomass of the non-mycorrhizal plants. *P*. *arundinacea* plants grew better in control substrate than in AMF substrate (Table 1). The biomass of D plants was unaffected by inoculation treatment in either substrate, N plants had the highest biomass when non-mycorrhizal (non-inoculated in control substrate) or when pre-inoculated in control substrate.

**Table 1:** Shoot dry weights of *M*. *sativa* and *P*. *arundinacea*.

Factors			Experimental Stage		
Plant	Substrate	Inoculation	D6	D12	N6
*M*. *sativa*	Control	NI	n.d.	0.88 (0.38) c	0.13 (0.05) b
		in-situ	0.30 (0.08) b	2.01 (0.30) b	0.43 (0.11) a
		pre	0.76 (0.09) a	2.71 (0.37) a	0.50 (0.16) a
	AMF	NI	0.93 (0.12) a	1.95 (0.39) b	0.49 (0.08) a
		in-situ	0.42 (0.07) b	1.64 (0.27) b	0.55 (0.19) a
		pre	0.75(0.11) a	1.81 (0.16) b	0.56 (0.09) a
**df**			4	5	5
**F-value**			41.2 ***	17.3 ***	8.4 ***
*P*. *arundinacea*	Control	NI	n.d.	2.74 (0.40) a	1.18 (0.19) a
		in-situ	0.86 (0.22) ab	2.76 (0.29) a	0.38 (0.09) b
		pre	1.16 (0.20) a	2.80 (0.18) a	0.89 (0.32) a
	AMF	NI	0.46 (0.21) c	1.08 (0.41) b	0.46 (0.20) b
		in-situ	0.56 (0.18) bc	0.70 (0.20) b	0.36 (0.19) b
		pre	0.41 (0.11) c	0.97 (0.22) b	0.30 (0.16) b
**df**			4	5	5
**F-value**			14.1 ***	52.3 ***	13.7 ***

The plants were grown in substrate with native AMF community (AMF) or sterilized control substrate (Control), non-inoculated (NI), inoculated in-situ (in-situ) or pre-inoculated (pre) with *R*. *irregularis* ‘Chomutov’. D6 and D12 are directly inoculated donor plants harvested after six or 12 weeks of cultivation, respectively; N6 are six-week-old neighboring plants. Values are g of shoot dry biomass, means of 4–6 replicates (SD), for exact replicate numbers see S12 Table. F-values are given according to one-way ANOVA with substrate × inoculation combination as factor.

*** P < 0.001. Means within column significantly different at P < 0.05 according to Tukey's test are marked by different letters; n.d. not determined.

## Discussion

This study presents, for the first time, experimental evidence for inoculation effects exceeding the directly inoculated plants. We found that

1. The inoculant spread into the root systems of neighboring plants;
2. The suppression of native AMF by inoculation, observed in the directly inoculated plants, was maintained in the neighboring plants.

### Establishment of the inoculant

Establishment of inoculated AMF in the roots of directly inoculated plants, observed in accordance with many previous studies, e.g. [10, 17, 20, 28], indicates that the inoculant successfully competed with native AMF in primary infection, intraradical spread and the formation of secondary infection units. This is facilitated by the inoculant's priority, either directly achieved by pre-inoculation or supported by high inoculum doses [23, 40], and does not necessarily mean establishment of a stable population of the inoculant at the site of inoculation. However, propagation of the inoculant into neigboring root systems, as documented in our experiment, requires also competitiveness in the spread of ERM and formation of infective propagules in soil. During these later stages of the symbiosis, the highly abundant inoculant may lose its competitiveness e.g. due to sanctioning by the host plant through preferential allocation of carbon to other AMF [41]. The comparable abundance of the inoculant in the directly inoculated plants and their neighbors of our experiment, however, do not indicate such a mechanism.

Inoculation did not introduce a new species in our experiment, as *R*. *irregularis* was also part of the native community. However, it introduced a new intraspecific genotype, which may ecologically and functionally have differed from the native genotypes [24, 42–43] and may have remained a separate genetic entity in case of vegetative incompatibility with the native genotypes [44–45]. The naturalization of an introduced organism occurs when it crosses the barrier of reproduction [46], and we argue that due to the obligate biotrophy of AMF, their successful reproduction is demonstrated by the ability to infect plants that were not in direct contact with the introduced propagules. In this respect, our experiment demonstrated naturalization of an AMF inoculant and establishment of a stable population, albeit in the artificial conditions of pot cultivation. Moreover, the documented horizontal spread by below-ground hyphal growth constitutes a vector of AMF dispersal [47]. Thus, the ability to infect neighboring plants by ERM indicates also dispersion potential, at least at small-scale level.

### Inoculation effects on native AMF

Concordantly with our results, inoculation was previously shown to increase the abundance of the inoculated AMF species and to reduce or even completely eliminate root colonization by other AMF species [10, 17–18, 28]. No complete exclusion of native AMF in the inoculated treatments of our experiment should be attributed to the employed detection system: The characterization of the AMF community was performed by Sanger sequencing of clones and certainly missed some low abundant taxa [48], which may also partly explain the relatively low diversity of the native community. However, the sensitivity of qPCR for low abundant taxa is even lower, as indicated by the failure to detect the *D*. *celata* clade. Thus, the employed approach concentrated on the relative abundances of the more abundant AMF community members, which may be expected to have larger impact on the functioning of the symbiosis.

Overdominance of one species occurs also in natural AMF communities and seems reversible by stochastic effects [49–50]. However, we cannot exclude that overdominance induced by inoculation may be permanent or even progressive and negatively impact on the functional parameters of the AMF community [51]. In our experiment, the inoculated and non-inoculated AMF communities became more similar in the neighboring plants than in the directly inoculated plants. However, this was rather due to shifts in the non-inoculated community than to changes in the inoculated communities that remained similar across the three stages. Therefore, we did not find any convincing evidence for short-term resilience in the AMF community composition after inoculation. This may be attributed to the specific conditions of the experiment: The tendency to increasing dominance of *R*. *irregularis* in the non-inoculated treatment indicates that this species was favored by the cultivation conditions and the effect of inoculation partly anticipated a trend that occurred also in the non-inoculated community. On the other hand, the presumably suitable conditions may also have progressively favored the inoculant alongside with progressive suppression of the other species, which was neither the case. The effect of inoculation was stable in the investigated time-frame, the AMF communities of the inoculated treatments were similar in the directly inoculated plants and in their neighbors.

A previous study suggesting short-term resilience of AMF communities after inoculation differed from the presented experiment in having inoculated the same intraspecific genotypes as already present in soil [22]. New genotypes may introduce new traits into a species' population and thus alter the population dynamics and the species' competitiveness within the AMF community. Additionally, genetically different conspecific isolates compete among each other [52], which is consistent with the abundance decline of native *R*. *irregularis* genotypes in the inoculated treatments of our experiment. Cultivated isolates of *R*. *irregularis* usually contain only one mtLSU haplotype [53–54], while mtLSU diversity is considerably higher at field sites [34, 55], probably due to cultivation-related bottlenecks and biases [34]. Consistently, we found only one mtLSU haplotype in the inoculated *R*. *irregularis* isolate, which had been long-term maintained in culture, and three haplotypes in the root-colonizing native population. Inoculation thus, on one hand, increased the genotype richness of the *R*. *irregularis* population by introducing a new genotype, but on the other hand probably also decreased its evenness by suppressing the genetically more diverse native population. Thus, the effects of inoculation were analogous at the interspecific and intraspecific level. However, we must be aware that intraspecific interactions are more diverse than interspecific interactions including also possible anastomosis formation and genetic exchange between genotypes [44–45]. Understanding them more in detail would require screening the populations with multilocus markers [56]. Yet, mtLSU haplotypes also represent functionally relevant categories and, in contrast to multilocus screenings, can be directly detected and quantified in experimental samples [34–35].

The pattern of inoculation effects was largely consistent between the two plant species and two modes of inoculation At community level, however, pre-inoculation tended to suppress the native AMF more than inoculation in situ (Fig 6). Different impact of the two modes of inoculation was anticipated due to previously documented suppression of colonization in roots already colonized by another AMF species [29–30, 57]. Interestingly, the differences between the pre-inoculated and in-situ inoculated AMF community persisted also in the neighboring plants. This indicates that priority effects are maintained also at the level of ERM infectivity, possibly by ensuring better excess to carbohydrates from the host plant [29, 57].

The selected system and the experimental approach strongly suggest that the native AMF community was composed of disturbance-tolerant ruderal AMF, which is also consistent with the phylogenetic placement of all the detected clades in Glomerales order [58]. It can be assumed that competition between the inoculant and the native community was more intense than if they were phylogenetically and functionally more distinct [2, 59]. However, ruderal systems such as early successional areas and fields are typical target sites of AMF inoculations, and inoculation is typically performed with easily cultivable, i.e. fast growing isolates originating from similar conditions as the target system [13]. For this reason, we may assume that the intensity of competition and shifts in AMF community composition encountered in our study are representative for systems where inoculation is usually performed.

### Functional impact of inoculation

Comparing the growth of non-mycorrhizal plants (i.e. non-inoculated plants in control substrate) and mycorrhizal plants (in all the other treatments) suggests that the two selected plants species differed in their response to mycorrhiza. The positive mycorrhizal growth response of *M*. *sativa* is consistent with some previous studies, e.g. [22, 60, 61], but its magnitude should not be overinterpreted in view of other possible microbial factors, which may have contributed. Despite the addition of microbial filtrates, rhizobial population was probably initially less abundant in the non-inoculated control substrate than in the other treatments, which may have influenced *M*. *sativa* growth along with mycorrhiza. In contrast to *M*. *sativa*, *P*. *arundinacea* growth throughout the experiment indicates accumulation of pathogens in the experimental system, which was especially harmful to the later planted N seedlings. Nevertheless, these considerations on the overall response to mycorrhiza in the two plant species do not discard comparisons of plant growth among the different inoculation treatments in AMF substrate. There, the microbial community was dominated by the microorganisms introduced with the original spoil bank soil, which constituted 80% of the cultivation substrate.

Positive effects of inoculation are usually related to higher root colonization [62]. Hence, it is not surprising that inoculation did not enhance plant growth in the AMF substrate, where the native AMF community alone ensured high root colonization levels. In contrast, growth of *M*. *sativa* was reduced by inoculation in-situ in comparison to both the non-inoculated and pre-inoculated treatment. It has been suggested that inoculation in-situ may suppress plant growth by increasing competition among the root-colonizing AMF [22]. In the roots of pre-inoculated plants, fungal competition may be less intense as further root colonization of already mycorrhized plants is regulated prior to AMF-root contact, via signals in root exudates [63]. However, in-situ inoculated *M*. *sativa* plants produced less biomass than pre-inoculated plants also in the control treatment with no native AMF. This suggests, alternatively, that the inoculant imposed high initial carbon costs on the host plant prior to starting supplying it with nutrients [64–65], which is consistent with the effect of pre-inoculation on seedling growth in the pre-cultivation stage. Altogether, plant growth did not indicate any inoculation-induced changes in the symbiotic efficiency of the AMF community, reported in some previous studies [17, 66]. However, our data suggest that pre-inoculation is more suitable to immediately promote plant growth than inoculation in-situ, possibly also because it suppresses interactions among the inoculant and native AMF during the root colonization process.

### Conclusions and outlooks

Propagation of inoculants and inoculation effects exceeding directly inoculated plants had been previously assumed both in relation to desired long-term improvement of symbiotic AMF communities [13, 67] and to possible negative consequences of inoculations [15], but a direct proof was missing. Our work represents the first experimental evidence for the spread of inoculated AMF and inoculation effects beyond the directly inoculated plants.

The main result was that root colonization by native AMF was similarly suppressed in the neighboring plants as in the directly inoculated plants. This was largely independent of host plant and inoculation procedure. Effects of inoculation on AMF diversity and mycorrhiza function, however, also depend on the inoculant's identity [10, 17, 19], which had not been manipulated in our experiment. Further studies should therefore focus this factor in order to confirm to which degree the findings depend on the inoculant's taxonomic identity and/or functional traits. The employed cultivation system is suitable for such a screening as it enables the elimination of interfering factors such as space heterogeneity of native AMF communities and climatic fluctuations. On the other hand, our experiment has not supported the idea of short-term post-inoculation resilience of AMF communities, and therefore shifts attention to long-term dynamics, which should be preferentially targeted in field conditions. Both directions must be followed in order to further improve our understanding of inoculation effects, which is critically needed for assessing the potential benefits and drawbacks of AMF inoculations.

## Supporting information

S1 Text Characterization of the diversity of native arbuscular mycorrhizal fungi. (PDF)

S1 Fig Phylogenetic analysis of the native arbuscular mycorrhizal fungal community. (PDF)

S2 Fig Phylogenetic analysis of *Rhizophagus irregularis* haplotypes. (PDF)

S1 Table Real-time PCR assays in nuclear ribosomal DNA. (PDF)

S2 Table Real-time PCR assays in mitochondrial ribosomal DNA. (PDF)

S3 Table Variation in root colonization. (PDF)

S4 Table Root colonization of the experimental plants. (PDF)

S5 Table Variation in the abundance of the inoculant *R*. *irregularis* Chomutov. (PDF)

S6 Table Variation in the proportion of the inoculant and the abundance of native *R*. *irregularis*. (PDF)

S7 Table Abundances of native *R*. *irregularis* and inoculated *R*. *irregularis* Chomutov. (PDF)

S8 Table Variation in Pielou's evenness index J', abundance of *Rhizophagus irregularis* and the sum of abundances of other AMF taxa. (PDF)

S9 Table Abundances of *C*. *claroideum*, 'uncultured Glomeraceae' and *F*. *mosseae*. (PDF)

S10 Table Variation in the abundances of C. claroideum, 'uncultured Glomeraceae' and F. mosseae. (PDF)

S11 Table Total dry weights of *M*. *sativa* and *P*. *arundinacea* D seedlings at planting into the experiment. (PDF)

S12 Table Numbers of evaluated replicates per experimental treatment. (PDF)
