# Supplementary material for: Inoculation effects on root-colonizing arbuscular mycorrhizal fungal communities spread beyond directly inoculated plants
Source: PLoS One. 2017 Jul 24;12(7):e0181525. doi: 10.1371/journal.pone.0181525 (PMC5524347; doi:10.1371/journal.pone.0181525)
Supplement: S1 Text — (PDF) [file pone.0181525.s001.pdf]

S1 Text: Characterization of the diversity of native arbuscular mycorrhizal fungi.

Characterization of the arbuscular mycorrhizal fungal community in the large subunit of nuclear ribosomal DNA (nrLSU)

Genomic DNA was extracted from deep-frozen root samples of six five-month-old cultures of medic in the spoil bank soil (1 sample per culture) using the DNeasy Plant Mini Kit (Qiagen) according to the manufacturer's instructions. Partial nuclear ribosomal DNA was amplified by nested PCR using previously published primer combinations [1]. The PCR's were performed in 20 μl. The reaction mixtures for both amplification steps contained 1× concentrated PCR buffer with KCl (Fermentas, Ontario, Canada), 0.2 mM of each dNTP, 2.0 mM of $MgCl_2$, 0.5 μM of each primer, 0.8 μg of BSA (Fermentas) and 1 unit of Taq DNA polymerase (Fermentas). A few nanograms of genomic DNA and 1000× diluted PCR products were used as the template for the first and second amplification step, respectively. The amplification was performed in an Eppendorf Mastercycler (Eppendorf, Hamburg) with the following thermal cycling conditions: 5 min of initial denaturation at 95°C followed by 38 (first step) or 35 (second step) cycles of 30 s denaturation at 95°C, 1.5 min annealing at 60°C (first step) or 63°C (second step) and 2 min elongation at 72°C, and a final extension step for 30 min at 72°C. The products of the parallel PCR reactions were pooled equimolarly, divided into two parts, cloned and further processed as previously published [2]. The inserts were re-amplified and sequenced (Macrogen, Inc., Seoul) using M13 universal primers. In total 72 clones were sequenced. The respective bacterial colonies were cultured overnight in liquid LB medium with ampicillin (100 μg ml-1), mixed with sterile glycerol to 13% glycerol concentration and stored at -80°C.

To identify the species composition of the AMF community under study, obtained sequences of the large subunit of nuclear ribosomal DNA (nrLSU) were compared with sequences from public databases using the online version of BLAST [3]. GenBank accessions with the highest level of similarity were aligned together with the obtained sequences using Clustal X [4]. The alignments were corrected manually in BioEdit [5]. Insertions and deletions were coded according to the simple gap coding method [6]. Maximum parsimony analysis was performed in PAUP* [7] using heuristic search with 100 random sequence addition replicates, saving no more than 100 trees with length ≥ 1 per replicate. Bootstrap analysis with 1000 replicates was performed using the same settings.

Characterization of the *Rhizophagus irregularis* population in the large subunit of mitochondrial ribosomal DNA (mtLSU)

The genetic variation of mtLSU of the native *R. irregularis* clade was characterized from the same root samples as nrLSU. The region was amplified by nested PCR using the primers RNL5 and RNL28a [8] in the first step, RNL29 [8] and GImt4510R in the second step, as previously described in detail [9]. The PCR products were further processed as described above for the nrLSU fragments including alignments and phylogenetic analysis of the exon sequences. The only difference was that several internal primers [9] were used for sequencing the clones.

References

1. Krüger M, Stockinger H, Krüger C, Schüssler A. DNA-based species level detection of Glomeromycota: one PCR primer set for all arbuscular mycorrhizal fungi. New Phytologist. 2009;183(1):212-23. doi: 10.1111/j.1469-8137.2009.02835.x.

2. Fehrer J, Krak K, Chrtek J. Intra-individual polymorphism in diploid and apomictic polyploid hawkweeds (*Hieracium*, Lactuceae, Asteraceae): disentangling phylogenetic

signal, reticulation, and noise. BMC Evolutionary Biology 2009;9:239. doi: 10.1186/1471-2148-9-239.

3. Altschul SF, Gish W, Miller W, Myers EW, Lipman DJ. Basic local alignment search tool. Journal of Molecular Biology. 1990;215(3):403-10. doi: 10.1006/jmbi.1990.9999.
4. Thompson JD, Gibson TJ, Plewniak F, Jeanmougin F, Higgins DG. The ClustalX windows interface: flexible strategies for multiple sequence alignment aided by quality analysis tools. Nucleic Acids Research. 1997;24:4876–82. doi: 10.1093/nar/25.24.4876.
5. Hall TA. BioEdit: a user-friendly biological sequence alignment editor and analysis suite. Nucleic Acids Symposium Series. 1999;41:95–8.
6. Simmons MP, Ochoterena H. Gaps as characters in sequence-based phylogenetic analyses. Systematic Biology. 2000;49(2):369-81. doi: 10.1093/sysbio/49.2.369.
7. Swofford DL. PAUP* Phylogenetic Analysis Using Parsimony (*and Other Methods). 4 ed. Sunderland, Massachusetts: Sinauer Associates; 2003.
8. Börstler B, Raab PA, Thiery O, Morton JB, Redecker D. Genetic diversity of the arbuscular mycorrhizal fungus *Glomus intraradices* as determined by mitochondrial large subunit rRNA gene sequences is considerably higher than previously expected. New Phytologist. 2008;180(2):452-65. doi: 10.1111/j.1469-8137.2008.02574.x.
9. Krak K, Janoušková M, Caklová P, Vosátka M, Štorchová H. Intraradical Dynamics of Two Coexisting Isolates of the Arbuscular Mycorrhizal Fungus *Glomus intraradices* Sensu Lato as Estimated by Real-Time PCR of Mitochondrial DNA. Applied and Environmental Microbiology. 2012;78(10):3630-7. doi: 10.1128/aem.00035-12.
