# Supplementary material for: Inoculation effects on root-colonizing arbuscular mycorrhizal fungal communities spread beyond directly inoculated plants
Source: PLoS One. 2017 Jul 24;12(7):e0181525. doi: 10.1371/journal.pone.0181525 (PMC5524347; doi:10.1371/journal.pone.0181525)
Supplement: S1 Fig — (PDF) [file pone.0181525.s002.pdf]

S1 Fig. Phylogenetic analysis of the native arbuscular mycorrhizal fungal community.

The analysis is based on sequences of partial large subunit of nuclear ribosomal DNA, the most parsimonious tree is presented. Bootstrap values higher than 50% are indicated by numbers above or below branches. The sequences obtained in the present study are given in bold.

* Sequences published by [1]; ** sequences published by [2].

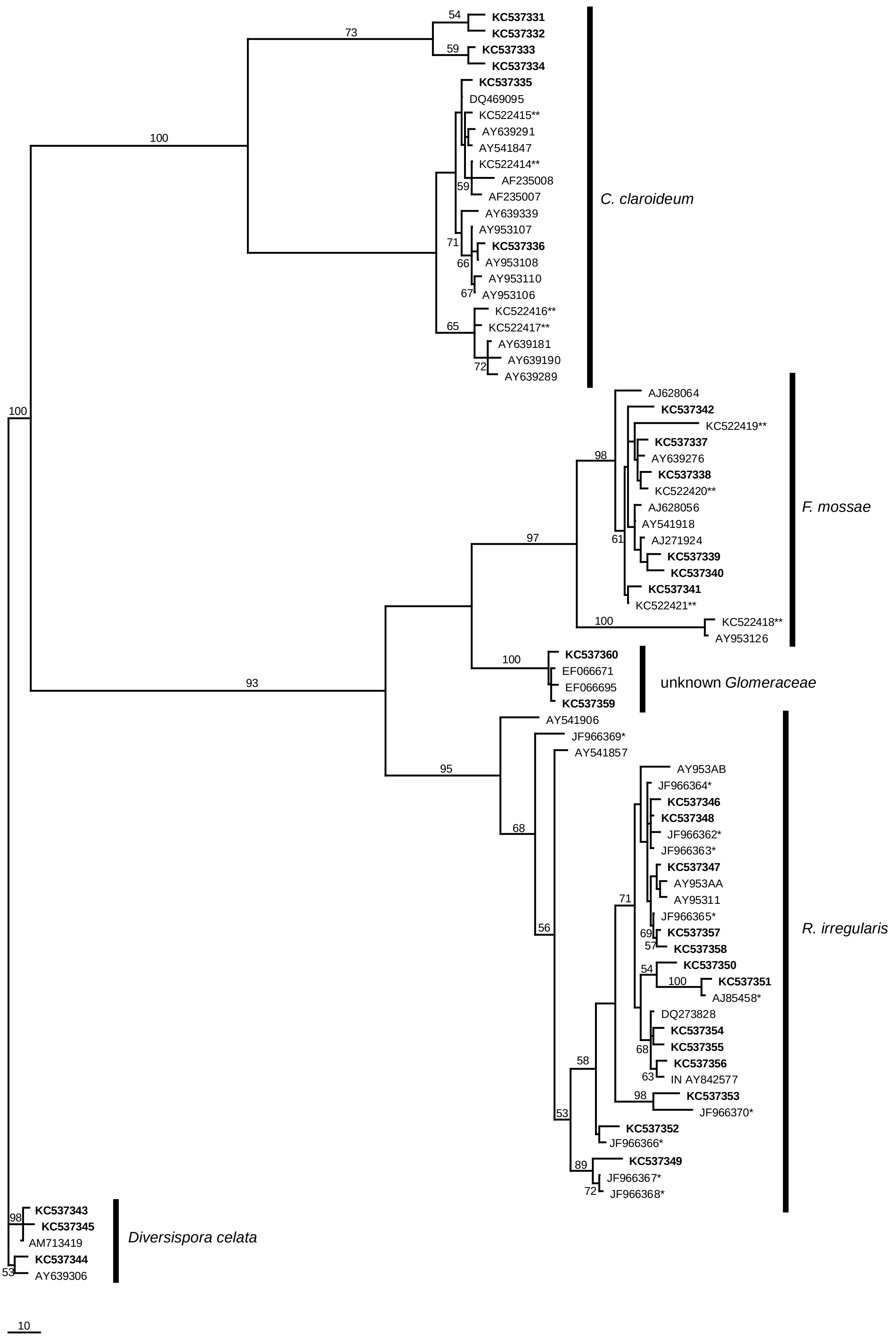

KC537331
KC537332
KC537333
KC537334
KC537335
DQ469095
KC522415**
AY639291
AY541847
KC522414**
AF235008
AF235007
AY639339
AY953107
KC537336
AY953108
AY953110
AY953106
KC522416**
KC522417**
AY639181
AY639190
AY639289
C. claroideum
AJ628064
KC537342
KC522419**
KC537337
AY639276
KC537338
KC522420**
AJ628056
AY541918
AJ271924
KC537339
KC537340
KC537341
KC522421**
KC522418**
AY953126
F. mossae
KC537360
EF066671
EF066695
KC537359
unknown Glomeraceae
AY541906
JF966369*
AY541857
AY953AB
JF966364*
KC537346
KC537348
JF966362*
JF966363*
KC537347
AY953AA
AY95311
JF966365*
KC537357
KC537358
KC537350
KC537351
AJ85458*
DQ273828
KC537354
KC537355
KC537356
IN AY842577
KC537353
JF966370*
KC537352
JF966366*
KC537349
JF966367*
JF966368*
R. irregularis
KC537343
KC537345
AM713419
KC537344
AY639306
Diversispora celata
10

References

1. Krak K, Janoušková M, Caklová P, Vosátka M, Štorchová H. Intraradical Dynamics of Two Coexisting Isolates of the Arbuscular Mycorrhizal Fungus *Glomus intraradices* Sensu Lato as Estimated by Real-Time PCR of Mitochondrial DNA. Applied and Environmental Microbiology. 2012;78(10):3630-7. doi: 10.1128/aem.00035-12.
2. Janoušková M, Krak K, Wagg C, Štorchová H, Caklová P, Vosátka M. Effects of Inoculum Additions in the Presence of a Preestablished Arbuscular Mycorrhizal Fungal Community. Applied and Environmental Microbiology. 2013;79(20):6507-15. doi: 10.1128/aem.02135-13.
