# Supplementary material for: Inoculation effects on root-colonizing arbuscular mycorrhizal fungal communities spread beyond directly inoculated plants
Source: PLoS One. 2017 Jul 24;12(7):e0181525. doi: 10.1371/journal.pone.0181525 (PMC5524347; doi:10.1371/journal.pone.0181525)
Supplement: S2 Fig — (PDF) [file pone.0181525.s003.pdf]

S2 Fig. Phylogenetic analysis of *Rhizophagus irregularis* haplotypes.

The analysis is based on exon sequences of large subunit of mitochondrial ribosomal DNA. One of the nine most parsimonious trees is presented. Bootstrap values higher than 50% are indicated by the numbers above or below branches. The sequences of the three *R. irregularis* haplotypes obtained in the present study are given in bold.

* *R. irregularis* isolate ‘Chomutov’ [1]; ** *R. irregularis* isolate PH5 [1].

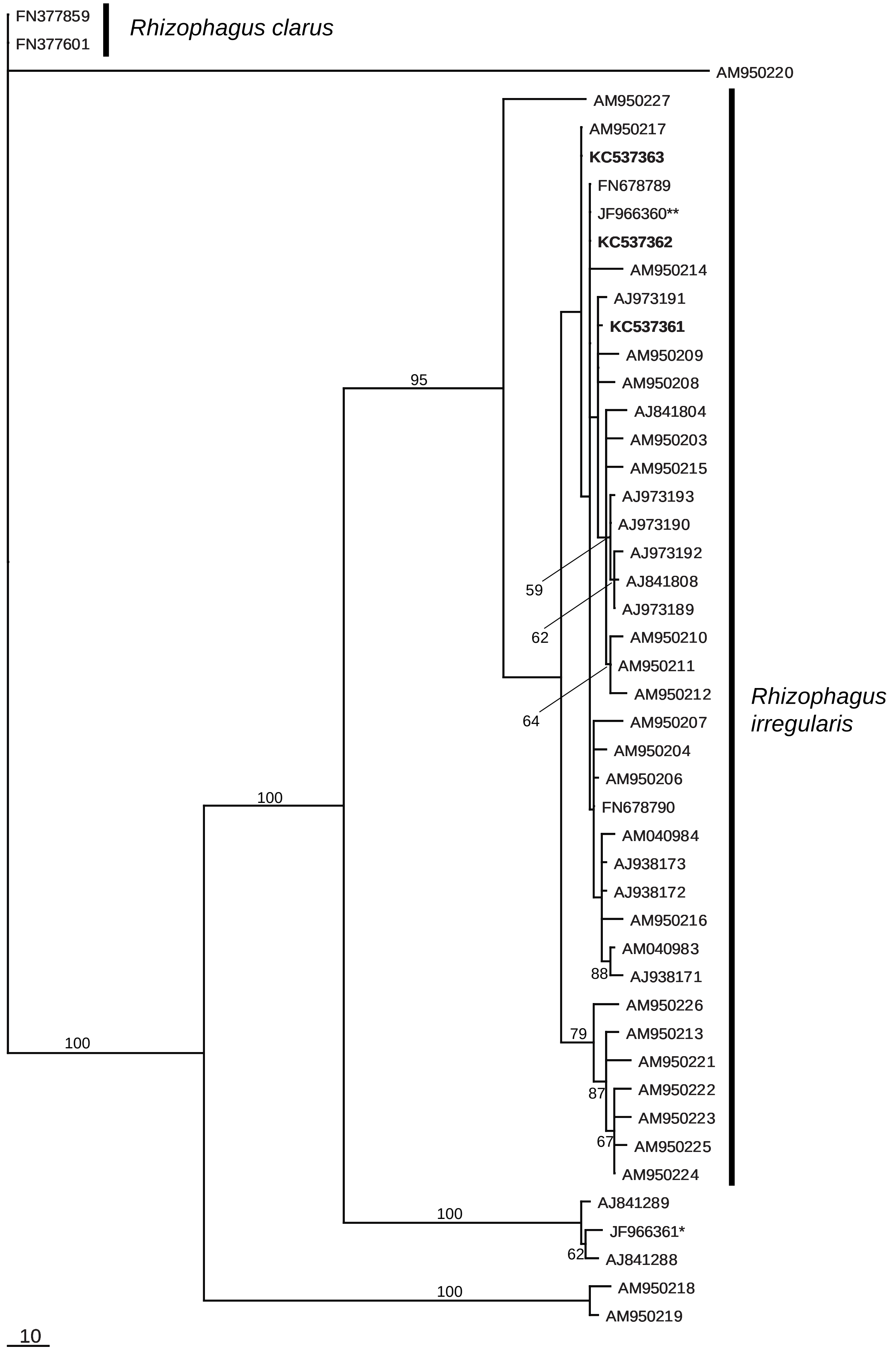

FN377859
FN377601
Rhizophagus clarus
AM950220
AM950227
AM950217
KC537363
FN678789
JF966360**
KC537362
AM950214
AJ973191
KC537361
AM950209
AM950208
AJ841804
AM950203
AM950215
AJ973193
AJ973190
AJ973192
AJ841808
AJ973189
AM950210
AM950211
AM950212
AM950207
AM950204
AM950206
FN678790
AM040984
AJ938173
AJ938172
AM950216
AM040983
AJ938171
AM950226
AM950213
AM950221
AM950222
AM950223
AM950225
AM950224
AJ841289
JF966361*
AJ841288
AM950218
AM950219
Rhizophagus irregularis
95
100
100
100
100
59
62
64
88
79
87
67
62
10

Reference

1. Krak K, Janoušková M, Caklová P, Vosátka M, Štorchová H. Intraradical Dynamics of Two Coexisting Isolates of the Arbuscular Mycorrhizal Fungus *Glomus intraradices* Sensu Lato as Estimated by Real-Time PCR of Mitochondrial DNA. Applied and Environmental Microbiology. 2012;78(10):3630-7. doi: 10.1128/aem.00035-12.
