# Supplementary material for: Inoculation effects on root-colonizing arbuscular mycorrhizal fungal communities spread beyond directly inoculated plants
Source: PLoS One. 2017 Jul 24;12(7):e0181525. doi: 10.1371/journal.pone.0181525 (PMC5524347; doi:10.1371/journal.pone.0181525)
Supplement: S1 Table — (PDF) [file pone.0181525.s004.pdf]

S1 Table. Real-time PCR assays in nuclear ribosomal DNA.

Target	Primer type	Primer name (primer sequence 5'→3')	Primer concn (µM)	Ta (°C)	Amplicon size (bp)	Amplification efficiency [a] (% ± SD)	Reference
RI	for<br>for<br>rev	GIX6-F351 (TTCGGGGCTACTTGTCTGAT)<br>GIX6-F351b (TTCGGGGCTACCTGTCTGGT)<br>GIX6-R424 (CCATCGACTTTGATAACCGTAA)	0.3<br>0.2<br>0.5	62	74	99.5 ± 3.1	[1]
CC	for<br>rev	GCX14-F300 (CTTTAGCGGAATGTGGTGGT)<br>GCX14-R431 (TAGCCTGTTCAAAGGCGAAT)	0.5<br>0.5	58	132	92.4 ± 2.4	[2]
FM	for<br>for<br>rev<br>rev	GMX2-F2 (CATACCAATGGGAAATCAACC)<br>GMX10-F (CGGGAAATCAACCTTTTGAG)<br>GMX2-R2 (CCGATGTTGACTAACTATACGAAAAA)<br>GMX10-R1 (CGATGTTGACTAACCGTACGAA)	0.25<br>0.25<br>0.25<br>0.25	60	112/<br>122	93.9 ± 1.8	[2]
UN	for<br>rev	UN-119F (TGTTGAAAGGGAAACGATTGA)<br>UN-195R (ACGCACGCCCACAAGTTC)	0.4<br>0.4	58	77	92.4 ± 2.8	Present study
DC	for<br>rev	DC-135F (ATTGAAGTCAGTCATGCTAGT)<br>DC-214R (GAGAAACGGTTTGACCCT)	0.4<br>0.4	62	80	93.7 ± 1.4	Present study

The assays were used for the quantification of the arbuscular mycorrhizal fungal clades *Rhizophagus irregularis* (RI), *Claroideoglomus claroideum* (CC), *Funneliformis mosseae* (FM), 'uncultured Glomeraceae' (UN) and *Diversispora celata* (DC).

[a] Amplification efficiency was calculated from at least three independent dilutions of plasmid templates.

References

1. Krak K, Janoušková M, Caklová P, Vosátka M, Štorchová H. Intraradical Dynamics of Two Coexisting Isolates of the Arbuscular Mycorrhizal Fungus *Glomus intraradices* Sensu Lato as Estimated by Real-Time PCR of Mitochondrial DNA. Applied and Environmental Microbiology. 2012;78(10):3630-7. doi: 10.1128/aem.00035-12.
2. Janoušková M, Krak K, Wagg C, Štorchová H, Caklová P, Vosátka M. Effects of Inoculum Additions in the Presence of a Preestablished Arbuscular Mycorrhizal Fungal Community. Applied and Environmental Microbiology. 2013;79(20):6507-15. doi: 10.1128/aem.02135-13.
