# Supplementary material for: Inoculation effects on root-colonizing arbuscular mycorrhizal fungal communities spread beyond directly inoculated plants
Source: PLoS One. 2017 Jul 24;12(7):e0181525. doi: 10.1371/journal.pone.0181525 (PMC5524347; doi:10.1371/journal.pone.0181525)
Supplement: S2 Table — (PDF) [file pone.0181525.s005.pdf]

S2 Table. Real-time PCR assays in mitochondrial ribosomal DNA.

Target	Primer type	Primer name (primer sequence 5'→3')	Primer concn (μM)	Ta (°C)	Amplicon size (bp)	Amplification efficiency [a] (% ± SD)	Reference
Inoculant	for	GI-CH-mtLSU-2545F (TGGATTTCTGGTTTTGTAGACG)	0.4	60	94	92.8 ± 1.4	[1]
	rev	GI-CH-mtLSU-2638R (ATTCTGCTTGTGGTTGTATTCC)	0.4				
Native genotypes	for	GI-PH5-mtLSU-219F (CAATTTGGCTGTATGCTGGA)	0.4	60	119	95.6 ± 3.1	[1]
	rev	GI-PH5-mtLSU-327R (GTGGTCGTTGAGGGGTTAAA)	0.4				

The assays were used for the quantification of the inoculant *R. irregularis* Chomutov and the native genotypes of *Rhizophagus irregularis*.

[a] Amplification efficiency was calculated from at least three independent dilutions of plasmid templates.

Reference

1. Krak K, Janoušková M, Caklová P, Vosátka M, Štorchová H. Intraradical Dynamics of Two Coexisting Isolates of the Arbuscular Mycorrhizal Fungus *Glomus intraradices* Sensu Lato as Estimated by Real-Time PCR of Mitochondrial DNA. Applied and Environmental Microbiology. 2012;78(10):3630-7. doi: 10.1128/aem.00035-12.
