# Supplementary material for: Inoculation effects on root-colonizing arbuscular mycorrhizal fungal communities spread beyond directly inoculated plants
Source: PLoS One. 2017 Jul 24;12(7):e0181525. doi: 10.1371/journal.pone.0181525 (PMC5524347; doi:10.1371/journal.pone.0181525)
Supplement: S3 Table — (PDF) [file pone.0181525.s006.pdf]

# S3 Table. Variation in root colonization.

| Factors and interactions | AMF substrate | | Control substrate | |
|---|---|---|---|---|
| | df | F | df | F |
| Plant species (A) | 1 | 8.70 ** | 1 | 4.95 * |
| Inoculation (B) | 2 | 1.24 | 1 | 3.12 |
| Stage (C) | 2 | 10.38 *** | 2 | 9.65 *** |
| A × B | 2 | 1.26 | 1 | 0.05 |
| A × C | 2 | 1.41 | 2 | 5.87 ** |
| B × C | 4 | 2.63 * | 2 | 2.36 |
| A × B × C | 4 | 0.81 | 2 | 0.10 |
| Residual | 84 | | 56 | |

ANOVA results are shown; separate ANOVAs were performed for the AMF substrate with native AMF community and the sterilized control substrate. The factor inoculation comprised three levels (no inoculation, in-situ inoculation, pre-inoculation) for the AMF substrate and two levels (in-situ inoculation, pre-inoculation) for the control substrate. Significance levels: * $P < 0.05$; ** $P < 0.01$; *** $P < 0.001$.
