# Supplementary material for: Inoculation effects on root-colonizing arbuscular mycorrhizal fungal communities spread beyond directly inoculated plants
Source: PLoS One. 2017 Jul 24;12(7):e0181525. doi: 10.1371/journal.pone.0181525 (PMC5524347; doi:10.1371/journal.pone.0181525)
Supplement: S4 Table — (PDF) [file pone.0181525.s007.pdf]

S4 Table. Root colonization of the experimental plants.

		M. sativa		*P. arundinacea*	
**Stage**	**Inoculation**	**AMF**	**Control**	**AMF**	**Control**
D6	NI	64 (10)	0	63 (15)	0
	in-situ	64 (14)	84 (10)	76 (20)	84 (10)
	pre	55 (20)	65 (26)	52 (10)	65 (26)
D12	NI	61 (13)	0	71 (15)	0
	in-situ	70 (19)	65 (8)	83 (7)	89 (12)
	pre	55 (16)	61 (18)	77 (15)	88 (4)
N6	NI	81 (12)	0	83 (13)	0
	in-situ	64 (8)	93 (2)	81 (16)	87 (14)
	pre	80 (15	91 (8)	81 (10)	93 (2)

Data show percentage of root colonization by arbuscular mycorrhizal fungi, means of 6 replicates (SD). The plants were grown in substrate with native AMF community (AMF) or sterilized control substrate (Control); non-inoculated (NI), inoculated in-situ (in-situ) or pre-inoculated (pre) with *R. irregularis* ‘Chomutov’. D6 and D12 are directly inoculated donor plants harvested after six or 12 weeks of cultivation, respectively; N6 are six-week-old neighboring plants.
