# Supplementary material for: Inoculation effects on root-colonizing arbuscular mycorrhizal fungal communities spread beyond directly inoculated plants
Source: PLoS One. 2017 Jul 24;12(7):e0181525. doi: 10.1371/journal.pone.0181525 (PMC5524347; doi:10.1371/journal.pone.0181525)
Supplement: S5 Table — (PDF) [file pone.0181525.s008.pdf]

**S5 Table. Variation in the abundance of the inoculant *R. irregularis* Chomutov.**

| **Factors and interactions** | **df** | **F** |
|---|---|---|
| Substrate (A) | 1 | 5.22 * |
| Plant species (B) | 1 | 3.89 |
| Inoculation (C) | 1 | 0.00 |
| Stage (D) | 2 | 1.54 |
| A × B | 1 | 6.26 * |
| A × C | 1 | 0.00 |
| B × C | 1 | 3.35 |
| A × D | 2 | 1.95 |
| B × D | 2 | 1.49 |
| C × D | 2 | 0.50 |
| A × B × C | 1 | 0.16 |
| A × B × D | 2 | 1.67 |
| A × C × D | 2 | 0.02 |
| B × C × D | 2 | 0.64 |
| A × B × C × D | 2 | 0.81 |
| Residual | 108 | |

The abundance was determined as copy numbers of mitochondrial ribosomal DNA. ANOVA results are shown; significance level: * $P < 0.05$.
