# Supplementary material for: Inoculation effects on root-colonizing arbuscular mycorrhizal fungal communities spread beyond directly inoculated plants
Source: PLoS One. 2017 Jul 24;12(7):e0181525. doi: 10.1371/journal.pone.0181525 (PMC5524347; doi:10.1371/journal.pone.0181525)
Supplement: S6 Table — (PDF) [file pone.0181525.s009.pdf]

S6 Table. Variation in the proportion of the inoculant and the abundance of native *R. irregularis*.

**Factors and interactions**	**Inoculant %**		**Native *R. irregularis***	
	**df**	**F**	**df**	**F**
Plant species (A)	1	5.46 *	1	0.00
Inoculation (B)	1	0.23	2	67.00 ***
Plant stage (C)	2	4.60 *	2	4.68 *
A × B	1	3.37	2	0.69
A × C	2	1.44	2	1.23
B × C	2	5.29 **	4	2.60 *
A × B × C	2	0.10	4	1.92
Residual	53		82	

Proportion of inoculated *R. irregularis* Chomutov at the total *R. irregularis* population (Inoculant %) is based on copy numbers of mitochondrial ribosomal DNA; abundance of the native *R. irregularis* genotypes was determined as copy numbers of mitochondrial ribosomal DNA. ANOVA results are shown; the factor inoculation comprised two levels (in-situ, pre-inoculation) for Inoculant % and three levels (no inoculation, in-situ, pre-inoculation) for native *R. irregularis*. Significance levels: * $P < 0.05$; ** $P < 0.01$; *** $P < 0.001$.
