# Supplementary material for: Inoculation effects on root-colonizing arbuscular mycorrhizal fungal communities spread beyond directly inoculated plants
Source: PLoS One. 2017 Jul 24;12(7):e0181525. doi: 10.1371/journal.pone.0181525 (PMC5524347; doi:10.1371/journal.pone.0181525)
Supplement: S7 Table — (PDF) [file pone.0181525.s010.pdf]

S7 Table. Abundances of native *R. irregularis* and inoculated *R. irregularis* Chomutov.

		Native *R. irregularis* AMF substrate		Inoculant AMF substrate		Inoculant Control substrate	
**Stage**	**Inoculation**	***MS***	***PA***	***MS***	***PA***	***MS***	***PA***
D6	NI	440 (238)	441 (128)	0	0	0	0
	in-situ	171 (136)	77 (58)	482 (310)	348 (187)	448 (315)	626 (404)
	pre	83 (70)	50 (51)	594 (304)	211 (79)	440 (308)	449 (223)
D12	NI	220 (101)	281 (112)	0	0	0	0
	in-situ	43 (35)	35 (24)	556 (139)	360 (120)	393 (141)	536 (284)
	pre	67 (32)	108 (68)	699 (273)	336 (55)	605 (215)	460 (302)
N6	NI	588 (501)	327 (75)	0	0	0	0
	in-situ	105 (70)	151 (129)	343 (131)	300 (22)	458 (178)	563 (222)
	pre	46 (49)	115 (42)	334 (124)	354 (95)	514 (139)	466 (112)

Abundances are given in thousands of copy numbers of mitochondrial ribosomal DNA in the roots of *Medicago sativa* (*MS*) or *Phalaris arundinacea* (*PA*) grown in substrate with native AMF community (AMF substrate) or sterilized control substrate; non-inoculated (NI), inoculated in-situ (in-situ) or pre-inoculated (pre) with the inoculant, *R. irregularis* 'Chomutov'. D6 and D12 are directly inoculated donor plants harvested after six or 12 weeks of cultivation, respectively; N6 are six-week-old neighboring plants. Data are means of 4-6 replicates (SD), see S12 Table for exact replicate numbers.
