# Supplementary material for: Inoculation effects on root-colonizing arbuscular mycorrhizal fungal communities spread beyond directly inoculated plants
Source: PLoS One. 2017 Jul 24;12(7):e0181525. doi: 10.1371/journal.pone.0181525 (PMC5524347; doi:10.1371/journal.pone.0181525)
Supplement: S8 Table — (PDF) [file pone.0181525.s011.pdf]

S8 Table. Variation in Pielou's evenness index J', abundance of *Rhizophagus irregularis* and the sum of abundances of other AMF taxa.

**Factors and interactions**	**J'**		***R. irregularis***		**other AMF taxa**	
	**df**	**F**	**df**	**F**	**df**	**F**
Plant species (A)	1	5.28 *	1	0.29	1	1.20
Inoculation (B)	2	71.18 ***	2	19.85 ***	2	45.98 ***
Plant stage (C)	2	2.79	2	1.93	2	3.64 *
A × B	2	3.28 *	2	1.38	2	3.19 *
A × C	2	0.80	2	3.08	2	3.98 *
B × C	4	5.83 ***	4	3.22 *	4	5.75 ***
A × B × C	4	1.71	4	2.33	4	0.09
Residual	82		82		82	

J' was calculated for the AMF communities based on copy numbers of nuclear ribosomal DNA of all taxa. The abundances were determined as copy numbers of nuclear ribosomal DNA, the abundance of 'other AMF taxa' was calculated by summing the abundances of *C. claroideum*, 'uncultured Glomeraceae' and *F. mosseae*. ANOVA results are shown; significance levels: * $P < 0.05$; ** $P < 0.01$; *** $P < 0.001$.
