# Supplementary material for: Inoculation effects on root-colonizing arbuscular mycorrhizal fungal communities spread beyond directly inoculated plants
Source: PLoS One. 2017 Jul 24;12(7):e0181525. doi: 10.1371/journal.pone.0181525 (PMC5524347; doi:10.1371/journal.pone.0181525)
Supplement: S9 Table — (PDF) [file pone.0181525.s012.pdf]

S9 Table. Abundances of *C. claroideum*, 'uncultured Glomeraceae' and *F. mosseae*.

		C. claroideum		**'uncult. Glomeraceae'**		***F. mosseae***	
**Stage**	**Inoculation**	***MS***	***PA***	***MS***	***PA***	***MS***	***PA***
D6	NI	19.7 (9.0)	5.9 (4.5)	14.2 (10.3)	6.2 (3.8)	64.9 (7.5)	72.6 (26.8)
	in-situ	14.7 (11.7)	4.0 (3.5)	7.8 (6.1)	2.7 (2.8)	40.1 (33.2)	20.7 (17.9)
	pre	9.0 (8.8)	2.3 (1.9)	2.3 (0.6)	3.0 (1.9)	7.2 (7.4)	5.5 (5.4)
D12	NI	12.4 (7.7)	3.0 (1.8)	23.0 (15.8)	56.1 (50.1)	12.2 (9.7)	21.8 (7.9)
	in-situ	5.5 (3.3)	0.5 (0.4)	7.0 (5.9)	1.6 (1.4)	4.3 (2.8)	11.0 (8.0)
	pre	6.9 (4.1)	0.6 (0.4)	9.1 (7.8)	3.4 (1.4)	2.4 (3.2)	23.9 (15.7)
N6	NI	7.3 (6.3)	1.5 (0.7)	7.0 (4.0)	6.4 (4.2)	36.1 (23.3)	44.4 (17.4)
	in-situ	21.3 (30.7)	2.1 (1.3)	5.4 (5.0)	1.7 (0.3)	28.5 (24.0)	30.3 (25.7)
	pre	3.8 (3.0)	1.4 (1.5)	6.5 (4.9)	0.6 (0.3)	10.2 (10.4)	16.5 (6.8)

Abundances are given in thousands of copy numbers of nuclear ribosomal DNA in the roots of *Medicago sativa* (*MS*) or *Phalaris arundinacea* (*PA*) grown non-inoculated (NI), inoculated in-situ (in-situ) or pre-inoculated (pre) with the *R. irregularis* ‘Chomutov’. D6 and D12 are directly inoculated donor plants harvested after six or 12 weeks of cultivation, respectively; N6 are six-week-old neighboring plants. Data are means of 4-6 replicates (SD), see S12 Table for exact replicate numbers.
