# Supplementary material for: Inoculation effects on root-colonizing arbuscular mycorrhizal fungal communities spread beyond directly inoculated plants
Source: PLoS One. 2017 Jul 24;12(7):e0181525. doi: 10.1371/journal.pone.0181525 (PMC5524347; doi:10.1371/journal.pone.0181525)
Supplement: S10 Table — (PDF) [file pone.0181525.s013.pdf]

S10 Table. Variation in the abundances of *C. claroideum*, 'uncultured Glomeraceae' and *F. mosseae*.

**Factors and interactions**	***C. claroideum***		**'uncultured Gl.'**		***F. mosseae***	
	**df**	**F**	**df**	**F**	**df**	**F**
Plant species (A)	1	80,30 ***	1	16,16 ***	1	9,11 **
Inoculation (B)	2	8,40 ***	2	37,27 ***	2	37,26 ***
Plant stage (C)	2	8,63 ***	2	12,01 ***	2	14,52 ***
A × B	2	0,27	2	4,97 **	2	1,95
A × C	2	0,72	2	0,99	2	9,20 ***
B × C	4	2,36	4	5,11 **	4	6,02 ***
A × B × C	4	0,38	4	3,87 **	4	1,12
Residual	82		82		82	

The abundances were determined as copy numbers of nuclear ribosomal DNA. ANOVA results are shown; significance levels: ** $P < 0.01$; *** $P < 0.001$.
