# Supplementary material for: Inoculation effects on root-colonizing arbuscular mycorrhizal fungal communities spread beyond directly inoculated plants
Source: PLoS One. 2017 Jul 24;12(7):e0181525. doi: 10.1371/journal.pone.0181525 (PMC5524347; doi:10.1371/journal.pone.0181525)
Supplement: S11 Table — (PDF) [file pone.0181525.s014.pdf]

S11 Table. Dry weights of *M. sativa* and *P. arundinacea* D seedlings at planting into the experiment.

	M. sativa	***P. arundinacea***
inoculated	29.4 (5.9)	10.6 (4.1)
not inoculated	38.7 (7.4)	20.8 (6.5)

The seedlings were either inoculated at germination with *R. irregularis* 'Chomutov' (for the pre-inoculation treatment) or left without inoculation (for the non-inoculated and in-situ inoculated experimental treatments). The total dry weights of plants are given in mg, values are means of five replicates (SD).
