# Supplementary material for: Inoculation effects on root-colonizing arbuscular mycorrhizal fungal communities spread beyond directly inoculated plants
Source: PLoS One. 2017 Jul 24;12(7):e0181525. doi: 10.1371/journal.pone.0181525 (PMC5524347; doi:10.1371/journal.pone.0181525)
Supplement: S12 Table — (PDF) [file pone.0181525.s015.pdf]

S12 Table. Numbers of evaluated replicates per experimental treatment.

		D6		D12		N6	
		MS	*PA*	*MS*	*PA*	*MS*	*PA*
AMF substrate	NI	6	6	6	6	6	6
	in-situ	6	6	**5**	**4**	**5**	**4**
	pre	6	6	6	**5**	6	**5**
Control substrate	NI	6	6	6	6	6	6
	in-situ	6	6	6	**5**	6	**5**
	pre	6	6	6	**4**	6	**4**

Medicago sativa (*MS*) or *Phalaris arundinacea* (*PA*) plants were grown in AMF substrate or Control substrate, non-inoculated (NI), inoculated in-situ (in-situ) or pre-inoculated (pre) with the *R. irregularis* 'Chomutov'. D6 and D12 are directly inoculated donor plants harvested after six or 12 weeks of cultivation, respectively; N6 are six-week-old neighboring plants. Decreased replicate numbers due to the dieback of N6 plants are highlighted in bold.
